# Cats’ and dogs’ welfare: text mining and topics modeling analysis of the scientific literature

**DOI:** 10.3389/fvets.2023.1268821

**Published:** 2023-10-12

**Authors:** Chrysa Adamaκopoulou, Beatrice Benedetti, Martina Zappaterra, Martina Felici, Naod Thomas Masebo, Annalisa Previti, Annamaria Passantino, Barbara Padalino

**Affiliations:** ^1^Department of Agricultural and Food Sciences, University of Bologna, Bologna, Italy; ^2^Department of Veterinary Sciences, University of Messina, Messina, Italy

**Keywords:** companion animals, canine, feline, well-being, behavior, systematic review, machine learning

## Abstract

Animal welfare is a field with increasing significance and has been raising huge concerns of the public and the political stage. Cats and dogs possess an important role in human life, but their welfare is not always secured from a legal aspect. This review aimed to describe the evolution and geographical distribution of “cats and dogs” and “puppies and kittens” welfare literature over the last 40 years, distinguish the main research topics studied and highlight gaps in knowledge. A search using Scopus® was performed with different search strings and predetermined filters as time range, language, and subject area. A total of 2,725 scientific literature records were retrieved but only the ones that referred to cats and dogs’ welfare aspects were retained. The final 1,775 records were processed through descriptive statistics, and text mining and topic analysis procedures were performed on their titles and abstracts. The results showed that the number of studies has been increasing, especially in Europe and North America. “Shelter” was the most frequent word, followed by “behavior,” “owner” and “adopt.” The nine topics that emerged from the analysis were breeding, stress and housing conditions, welfare and pain assessment, public health, shelter management and euthanasia, behavioral problems, health issues and management, human-animal interaction, and owners’ and veterinarians’ perceptions. While stress and housing conditions, public health, and owners’ and veterinarians’ perceptions were the most studied topics, human-animal interaction was the least studied. This review confirmed the increasing research and interest in cats’ and dogs’ welfare and showed gaps in knowledge where further studies are needed.

## 1. Introduction

Public concerns regarding the ethical treatment of animals have been growing over the years. This increased interest has led to the establishment and development of animal welfare science (1). According to Broom (2) the definition of welfare states ‘The welfare of an individual is its state as regards its attempts to cope with its environment.’. Animal welfare science can be characterized as one of the most complicated and inclusive fields in biology (3). It raises a variety of concerns that involve the fundamentals of life such as freedom from pain and injury, water and food supply, and shelter. These concerns can be grouped into three main headings that focus on proper biological function, balanced emotional state, and expression of natural behavior (4). An animal must be in good physical and mental health status to reach a balanced emotional state (5).

Animal welfare has been a subject of political interest for several decades (6, 7). Legislation on livestock animal welfare involves a highly strict framework on welfare on-farm, during transportation and slaughter (8) and they are often based on research-based evidence (9–12). On the contrary, in the case of cats and dogs, regulation worldwide has been slower to develop, both in terms of topics covered and specificity. In regions such as the United States and the South Wales State in Australia, legislation has the minimal requirements regarding dog and cat keeping and everyday handling. In fact, most of the time, the legislation is just between the lines of general anti-cruelty and animal welfare statutes (13). In Europe, the legislation only focuses on transportation and veterinary controls, making all the other aspects of pet welfare to be monitored by a national regulation system that differentiates from state to state (8). Cats and dogs hold a significant place in people’s daily lives and are considered members of their families providing not only companionship but also serving as a source of affection and emotional attachment (14). Dogs have expanded their role from being companions to providing aid as guides and assistants for people with disabilities (15, 16). As pets play a crucial role in providing companionship, the changing human lifestyles and demands can harm their well-being (17). It seems easy to presume the idea that pets are treated with respect as companion animals and their “good welfare” is granted, but there is not much evidence to confirm this belief (18). In fact, despite this assumption, there remain significant concerns regarding pet welfare (18, 19). One of the biggest issues undermining the welfare of dogs and cats is pet overpopulation and the burden on animal shelters. In the US, more than 3.5 million entries in shelters (including both dogs and cats) were recorded in 2019 (20). In Europe, there is currently no official data on the number of dogs and cats in shelters, but it is estimated that there are about 100 million abandoned pets in total, including not only those in shelters but also stray dogs and cats (21). Stray dogs are a major problem in several areas of southern and eastern Europe and a major public health concern, increasing the risk of aggression toward humans and other livestock and the transmission of rabies (22). Another area of animal welfare concern is the breeding of brachycephalic breeds of dogs and cats with brachycephalic (shortened, flattened) head structures, which also raises ethical concerns due to the associated health problems (23, 24). Furthermore, recently there has been an alarming rise in pet obesity, primarily due to limited access to exercise and excessive food consumption, resulting in severe health issues (24). A significant number of dog owners mistakenly believe that an overweight body condition is ideal for their pets (25). On the other hand, some pet owners are interested in feeding a plant-based diet, but vegetarian and vegan diets have been considered contraindicated in cats (26, 27). Finally, when humans anthropomorphize animals, they attribute to them their traits, emotions, or intentions, and this attitude may compromise pet welfare too (28).

The need for mandatory legislation to establish common policies and address inadequate legal systems must therefore be undertaken immediately (29). Fortunately, new regulations to protect companion animals’ welfare will be issued shortly in Europe (30) but there is a need for research-based evidence. Starting from these considerations, this review aimed to examine in detail this research field by using text mining and topic analysis techniques. The goal of text mining analysis is to identify the most important words within the text and topic modeling is a tool to uncover the structure of meaningful topics among collections of records as well as to discover hidden textual patterns (31). Through this analysis, this review seeks to extract valuable insights from a vast amount of scientific literature enabling the analysis of different topics within the field, tracking their evolution over time, and identifying any gaps in knowledge.

## 2. Materials and methods

### 2.1. Data Set

**Figure 1:**
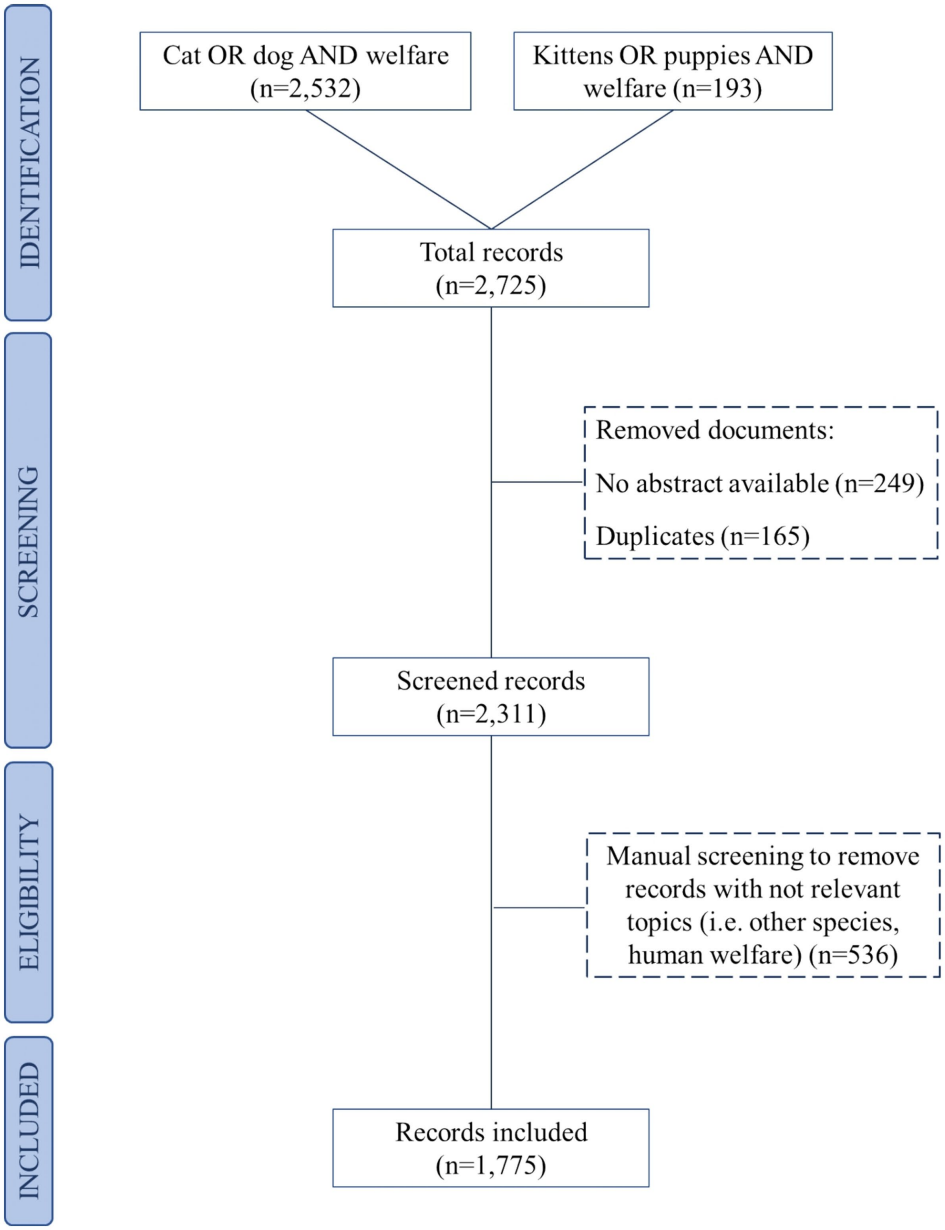
Flow chart representing scientific literature search and each step of selection of the scientific literature records on dogs’ and cats’ welfare. The number of the excluded records and their reason for exclusion from the study are represented by the dashed lines. Not-relevant species were every species different from dogs and cats.

A literature search protocol using Scopus®, the abstracts and citation database of Elsevier©, was set up to identify the peer-reviewed records that covered the topic of “cats and dogs’ welfare” and “puppies and kittens’ welfare.” The search was performed during May and June 2023. The research was refined based on the year of publication (from 1980 to 2023), scientific area (Veterinary and Agricultural and Biological Sciences topics), article type (review and scientific article), and language (English). The first search string, “cat OR dog AND welfare,” retrieved 2,532 records, while the second search string, “puppy OR kitten AND welfare,” retrieved 193 records. Setting these conditions, the produced records were 2,725. These records were inserted in an electronic Excel workbook (Microsoft Excel®, v16.0, Redmond, WA, United States) to perform further screening and analysis. The Excel spreadsheet organized the information in a tabular format, where each record was represented in a row, and the record’s information was organized in columns. The information in each column included title, authors, affiliations, abstracts, year of publication, type of record (e.g., article or review), and source of publication (i.e., name of the journal). After that, the elimination of the duplicates was performed since the same records could have been included in both search strings conducted. Starting from the original 2,725 records, an automatic exclusion of the records that had no abstract available was then performed for the construction of the final spreadsheet. After that, a further manual exclusion was performed by the four reviewers (CA, MZ, MF, and BP). The criteria for the manual exclusion were wrong topics, such as records about human welfare that referred to how pet companionship increases the well-being of humans and records that focused on other species (e.g., cattle). During the latter screening, the scientific records were categorized based on the relevant species (i.e., dogs, cats, dogs, and cats). The final number of records included was 1,775. Results of the systematic scientific literature search and the subsequent automatic and manual screening of records are represented schematically in Figure 1. Descriptive statistics were performed on the selected records to profile the scientific corpus (i.e., authors, country of the corresponding author, title of the paper, abstract, year and journal of publication) based on information recorded from the Scopus® database. Descriptive statistics was also performed based on the species (i.e., dogs, cats, or both) included in the scientific literature records. Pivot tables were made to count the number of records per year and to highlight the most represented nationality and regions in the document corpus. The nationality of each document was derived based on the affiliation of the corresponding author/first author.

### 2.2. Text mining

To conduct text mining analysis, a separate Excel sheet was created with two specific columns: “doc_id,” with the progressive numeration of the 1,775 scientific literature records, and “text” which contained the abstracts of the retained records. Text mining analysis involved converting the text into a numerical representation to identify important patterns within the data corpus. Since some words in the corpus of records were spelt in both American and British English, the authors decided to standardize the corpus of documents using only British English. The text mining analysis was performed in the R studio environment using a combination of functions in the packages “tm,” “snowballC,” “ggplot2,” “dplyr” and “tidyverse.” Text mining was performed considering the titles and abstracts of the 1,775 records. The pre-processing steps that the researchers followed involved what was reported in the literature (32). Namely:

- Convert the text to lowercase: All the capital letters inside the corpus were converted into lowercase letters.
- Removal of strange symbols and fonts: Symbols and fonts such as “@,” “/” or “*” were removed and replaced by white space.
- Removal of punctuations: Punctuations in the corpus were removed and replaced by white spaces.
- Exclusion of certain characters: punctuation, blanks, and numerical digits.
- Exclusion of “stop words.” These frequently used words, while common in the language, do not provide specific information about the content of the document. In the case of this review the researchers decided to remove as stopwords the following words: “dog,” “dogs,” “cat,” “significance,” “significant,” “significantly,” “group,” “groups, “test,” “animal,” “animals,” “study,” “studies,” “cats,” “welfare,” “well-being,” “research,” “researches,” “will,” “control,” “data,” “different.”
- Removal of numbers: Numbers were removed and replaced by white spaces.
- Removal of extra white spaces: Extra white spaces that occurred from previous steps were removed.
- The application of a stemming algorithm. This involves reducing words to their root forms, also known as tokenization, and helps to avoid counting the same word multiple times when it appears in different grammatical forms (e.g., “management” and “managerial” become “manag”). Stemming helps to standardize the representation of words and allows for a more accurate analysis of word frequencies and associations.

Afterward, a matrix was built containing along the rows and the terms along the columns, the so-called document-term matrix and a term frequency-inverse document frequency (TF-IDF) technique were used. The TF-IDF technique, employed to assign a relative weight to words (33), considers the frequency of a term within a document while considering how widely it is used across the entire collection of records. This adjustment reflects the importance of a word in the overall context of the document set. To identify the most important words, a threshold of TF-IDF value greater than or equal to 13 was used. These highly relevant words were then represented as a histogram, visually displaying their frequencies. Additionally, a word cloud was created.^1^ In this word cloud, the size of each word is proportional to its TF-IDF value. A larger character size indicates a higher TF-IDF value, highlighting the words that are more significant in the collection of records. Associations among the most frequent words (TF-IDF *≥* 13) and all the corpus terms were determined. The grade of correlation was set *≥*0.2 and associations were identified by measuring the frequency with which two words appear together. In particular, the correlation is 1 if two words are always together and − 1 if they are never together.

### 2.3. Topic analysis

The approach used for the topic modeling analysis in this review was the Latent Dirichlet Allocation (LDA). It is a probabilistic model based on the intuition that a single topic can be described as a multinomial distribution of words and a single document can be described as a multinomial distribution of latent topics (34). The words used in the topic analysis were those contained in the titles and abstracts of the 1,775 scientific literature records after pre-processing and text mining steps. The LDA function was used with the Gibbs sampling option of the “topic models” package in R (35). The LDA function returns a list of objects, which was then passed to the function ‘topics’ to create a table where each record is matched with one of the topics. We decided *a priori* to look at 6 and 9 topics for the topic analysis, and with the consensus among the researchers, the most indicative was chosen. Then, the resulting topics were ranked according to the cumulative probability of the first 15 words of each topic. The individual topics were visualized in a bar histogram representation with the probabilities of the first 15 words within each topic (beta values) and the authors attributed a name to each topic as suggested in the literature (36).

## 3. Results

### 3.1. Descriptive statistics

**Figure 2:**
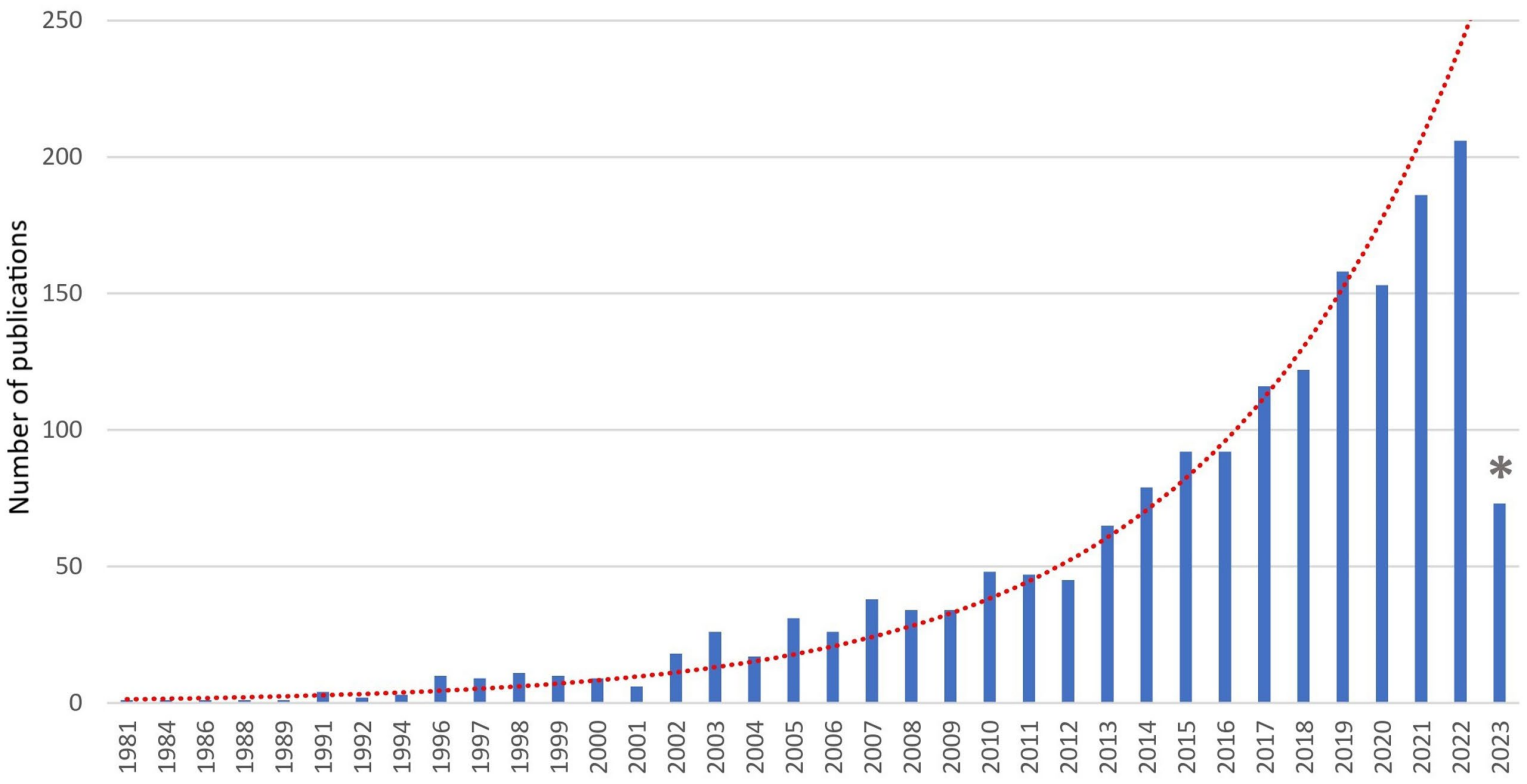
Peer-reviewed scientific literature records (*n* = 1,775) on the welfare of cats, dogs, puppies, and kittens from 1980 to 2023. The dashed line represents the trend over the years. The asterisk on the year 2023 indicates that results for that year are related to the period from January to March.

The number of publications per year from 1980 to 1994 was fewer than 10 records, whereas there was a significant increase in the number of records from 2005 to the present year (Figure 2).

**Figure 3:**
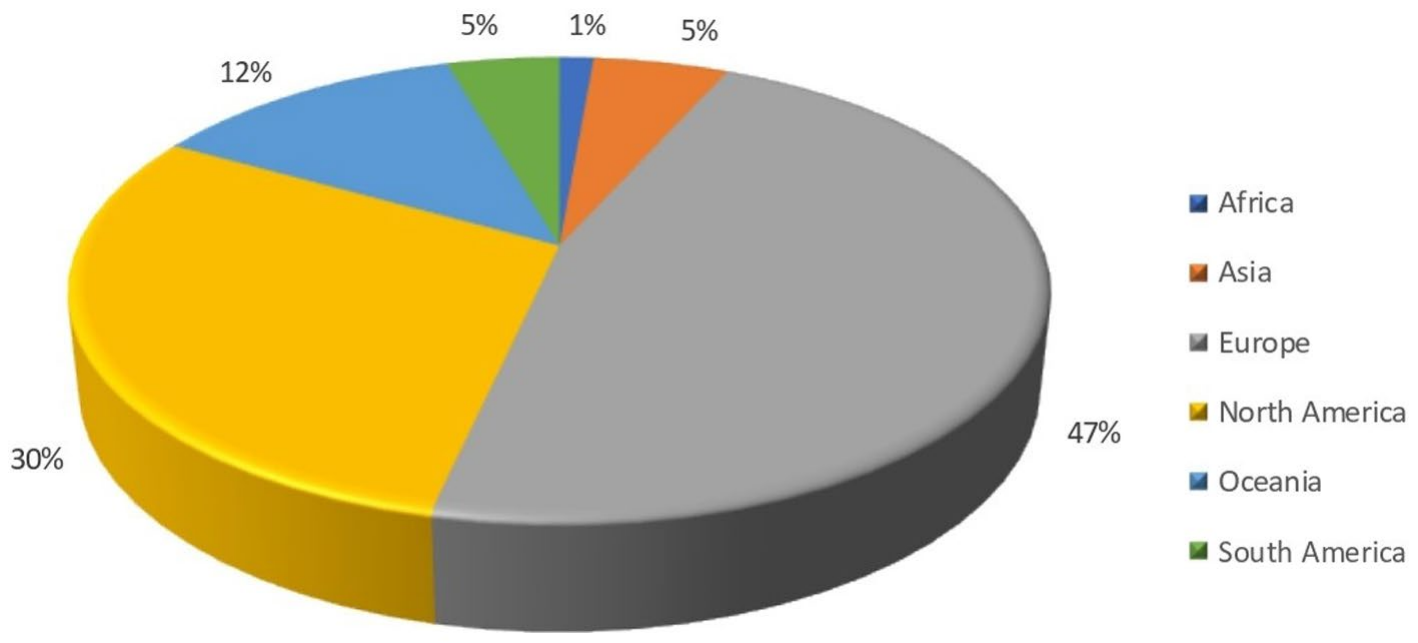
Pie chart depicting the distribution of the 1,775 scientific literature records selected for inclusion per regions and subregions, represented by their respective percentages.

**Figure 4:**
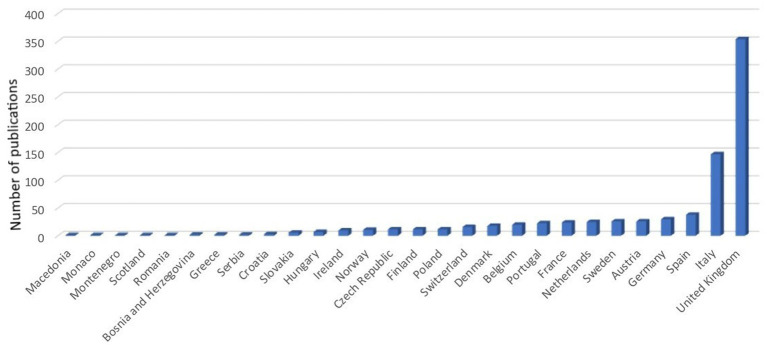
Distribution by European country of peer-reviewed scientific literature records (n = 1,775) on the welfare of cats, dogs, puppies, and kittens based on the nationality of the corresponding/first authors that are published from 1980 to 2023.

Nearly half of the identified records (47%) had corresponding or first authors from Europe, making it the most prominent region in terms of authorship. North America accounted for 30% of the records, making it the second most important region researching companion animal welfare topics. Oceania, Asia, South America, and Africa had progressively lower percentages of records, with 12, 5, 5, and 1%, respectively. The results are shown as a pie chart in Figure 3. Figure 4 shows instead the graph of European nations with the most records.

Dogs were the species with the highest number of records (1,031/1775, 58.1%). There were 455/1775 (25.6%) records for cats and 289/1775 (16.3%) records relating to both species.

### 3.2. Text mining

**Figure 5:**
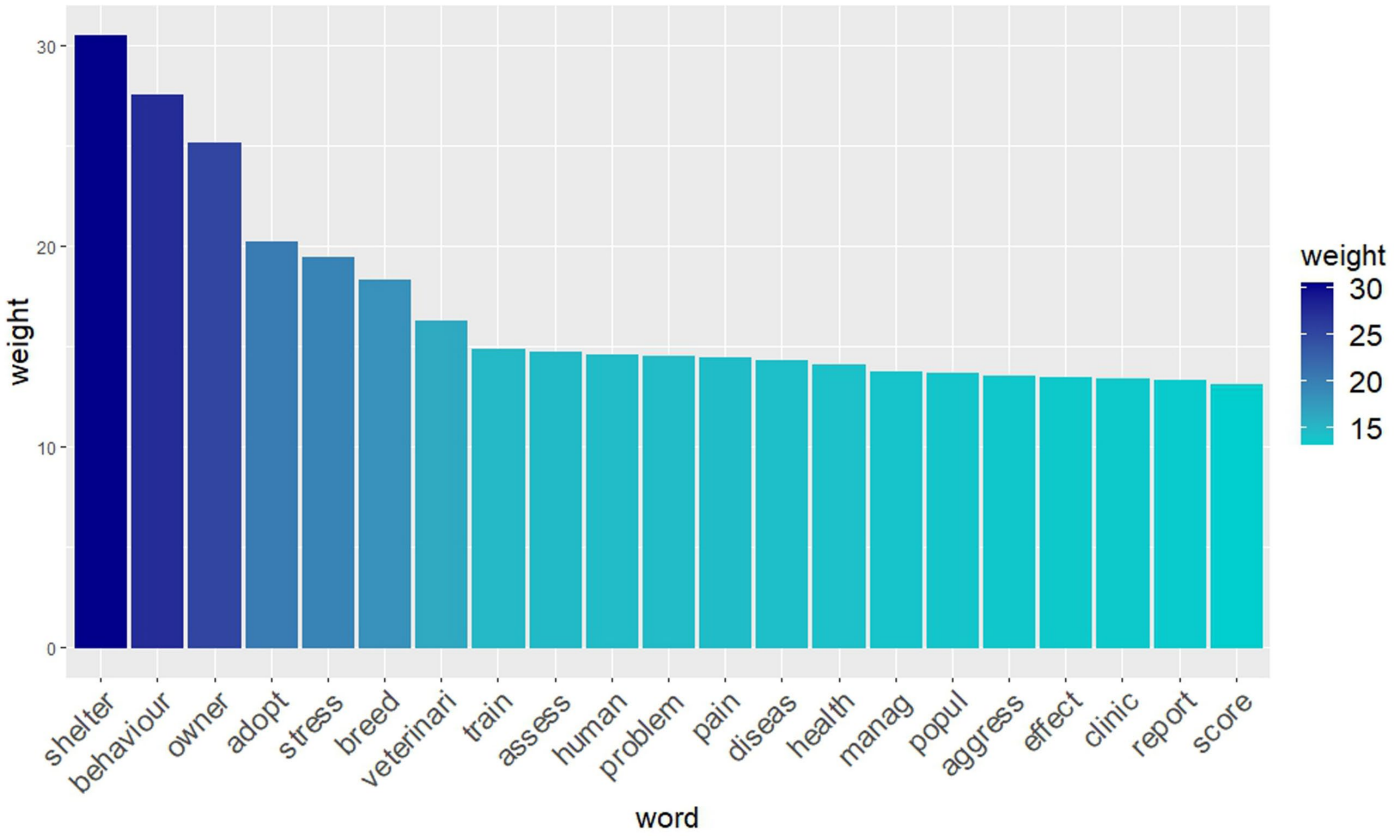
Histogram illustrating the most frequent words (i.e., words with term frequency-inverse document frequency (TF-IDF) values ≥13) and the weight of the 1,775 records included in the study.

**Figure 6:**
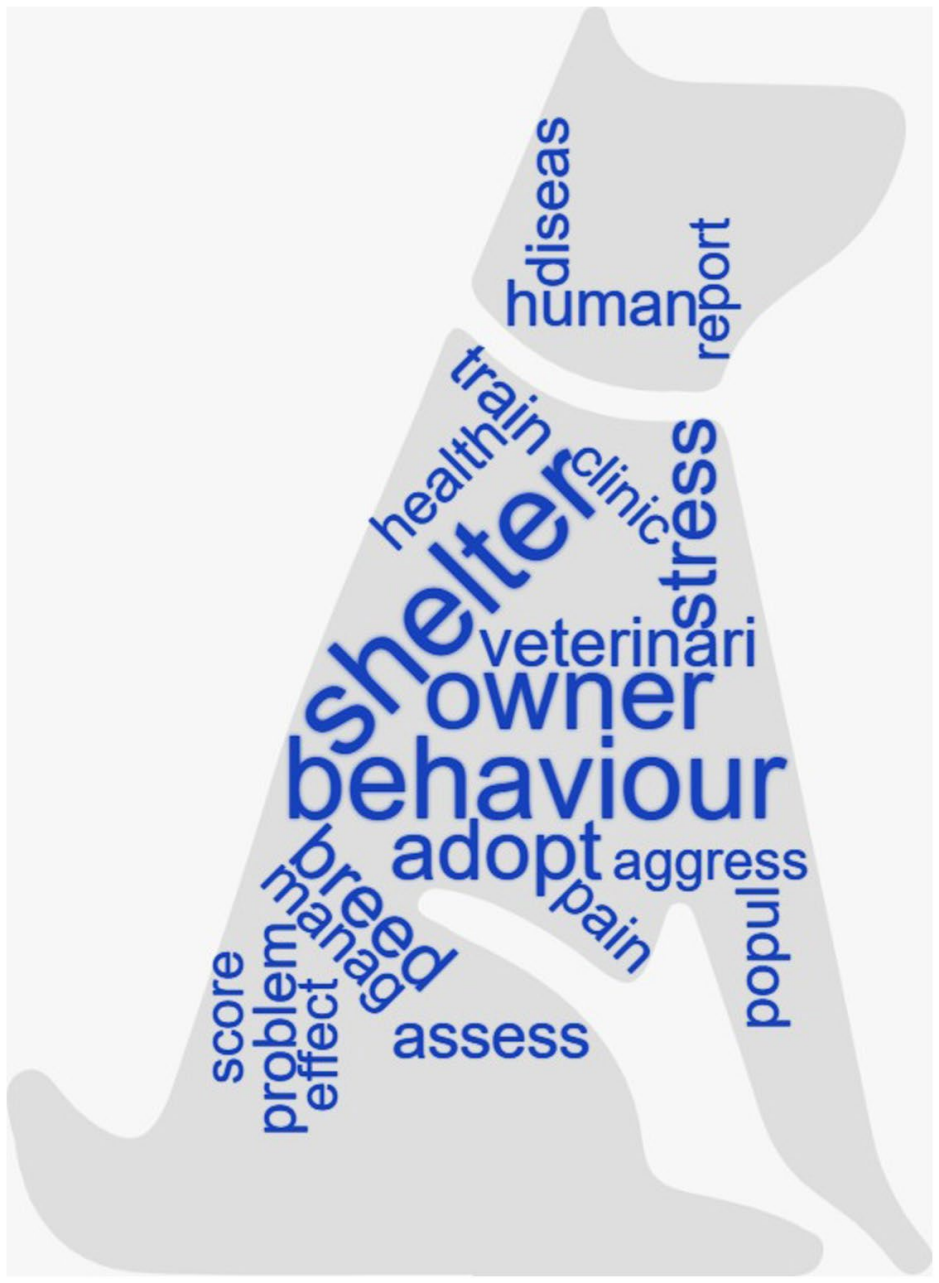
Word cloud with the most frequent words (i.e., words with term frequency-inverse document frequency (TF-IDF) values ≥13) of the 1,775 records included in the study. The words with larger font are the ones with higher weight.

The most frequent words with a weight over 13 (TF-IDF ≥ 13) are shown as a histogram in Figure 5. A word cloud with the most frequent words is represented in Figure 6 in which the size of the font is proportional to the TF-IDF of every word. A correlation coefficient of 0.2 was discovered between the most important words (with a TF-IDF score of 13 or higher) and the remaining words in the matrix. These correlations are presented in Table 1. No significant correlation (with correlation grade ≥ 0.2) with other words was shown by the words “behavior,” “human,” “manag,” “effect” and “report.”

**Table 1:** Correlation between the most relevant words (i.e., words with term frequency-inverse document frequency (TF-IDF) values ≥13) and the other words present in the corpus.

Words (TF-IDF ≥ 13)	Associated words (correlation ≥0.2)
Assess	tool (0.35), valid (0.34), reliabl (0.33)
Breed	pedigree (0.30), brachycephal (0.28), terrier (0.25), club (0.25), select (0.25), breeder (0.23), genet (0.22), bull (0.21), phenotyp (0.21), inherit (0.20)
Pain	analgesia (0.41), analges (0.34), scale (0.33), acut (0.31), chronic (0.22), challeng (0.21), advanc (0.20)
Shelter	enter (0.33), intak (0.24), stay (0.23), euthanasia (0.21), euthan (0.20)
Train	reinforc (0.43), trainer (0.35), punish (0.28), method (0.26), obedi (0.26), learn (0.23)
Adopt	return (0.26), characterist (0.25), color (0.21), coat (0.21), length (0.20)
Disease	infecti (0.24), preval (0.24), transmiss (0.24), infect (0.23), diagnosi (0.20)
Health	public (0.22)
Owner	questionnaire (0.26)
Problem	excess (0.26), destruct (0.21)
Stress	level (0.30), cortisol (0.25), stressor (0.21)
Veterinarian	surgeon (0.30), care (0.26), veterinarian (0.24), patient (0.23), client (0.21), medicin (0.21), practic (0.21), practition (0.21), nurs (0.20)
Agress	toward (0.30), fear (0.24)
Clinic	sign (0.27)
Popul	freeroam (0.34), dynam (0.27), densiti (0.22), roam (0.21), capac (0.20)
Score	qualiti (0.23), centr (0.20)

The grade of correlation was set ≥0.2.

### 3.3. Topic analysis

**Figure 7:**
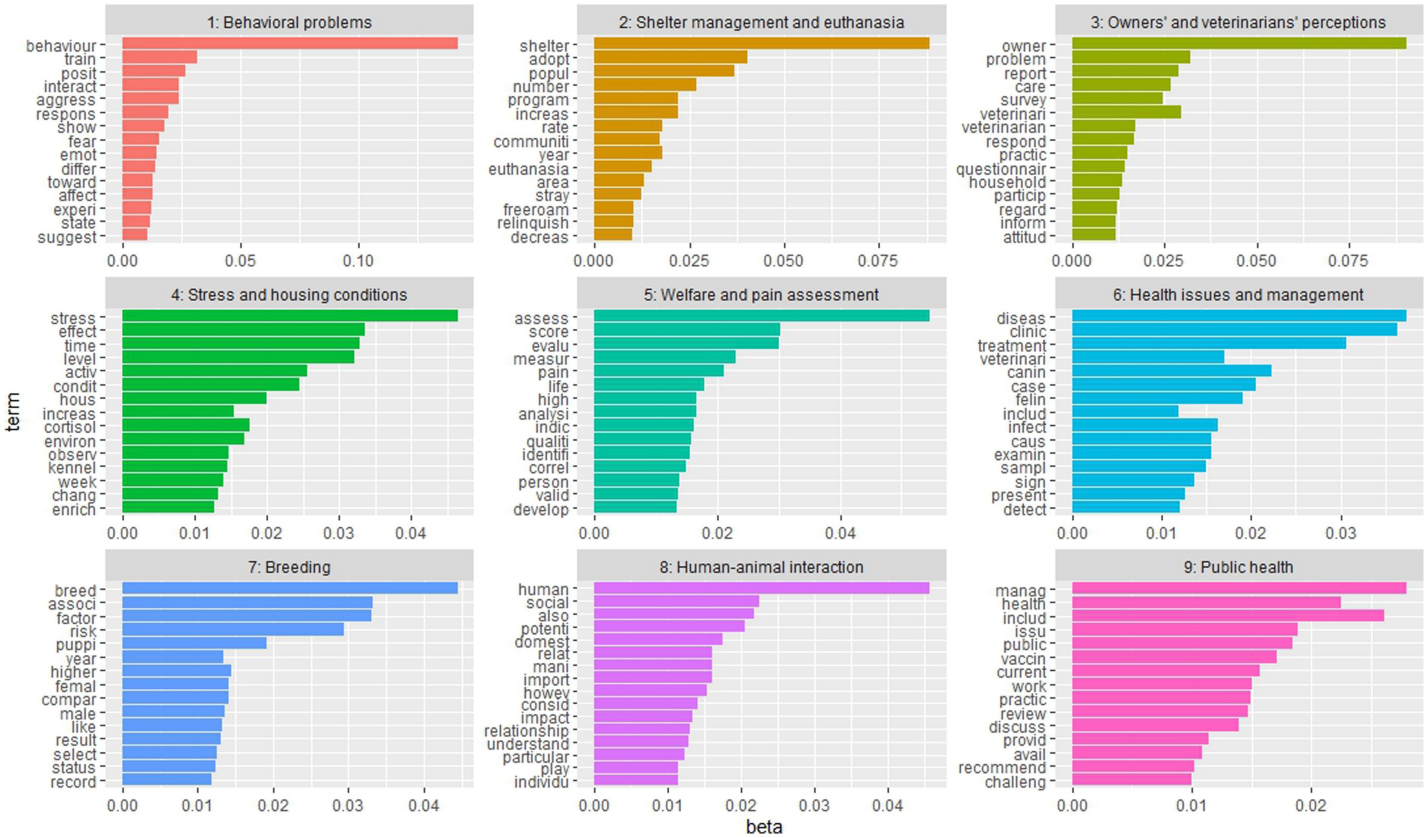
Histograms with the most frequent 15 words (terms) for the 9 topics of the 1,775 scientific literature records included in the study. The “beta” indicates the relative probability of each word belonging to each topic.

**Figure 8:**
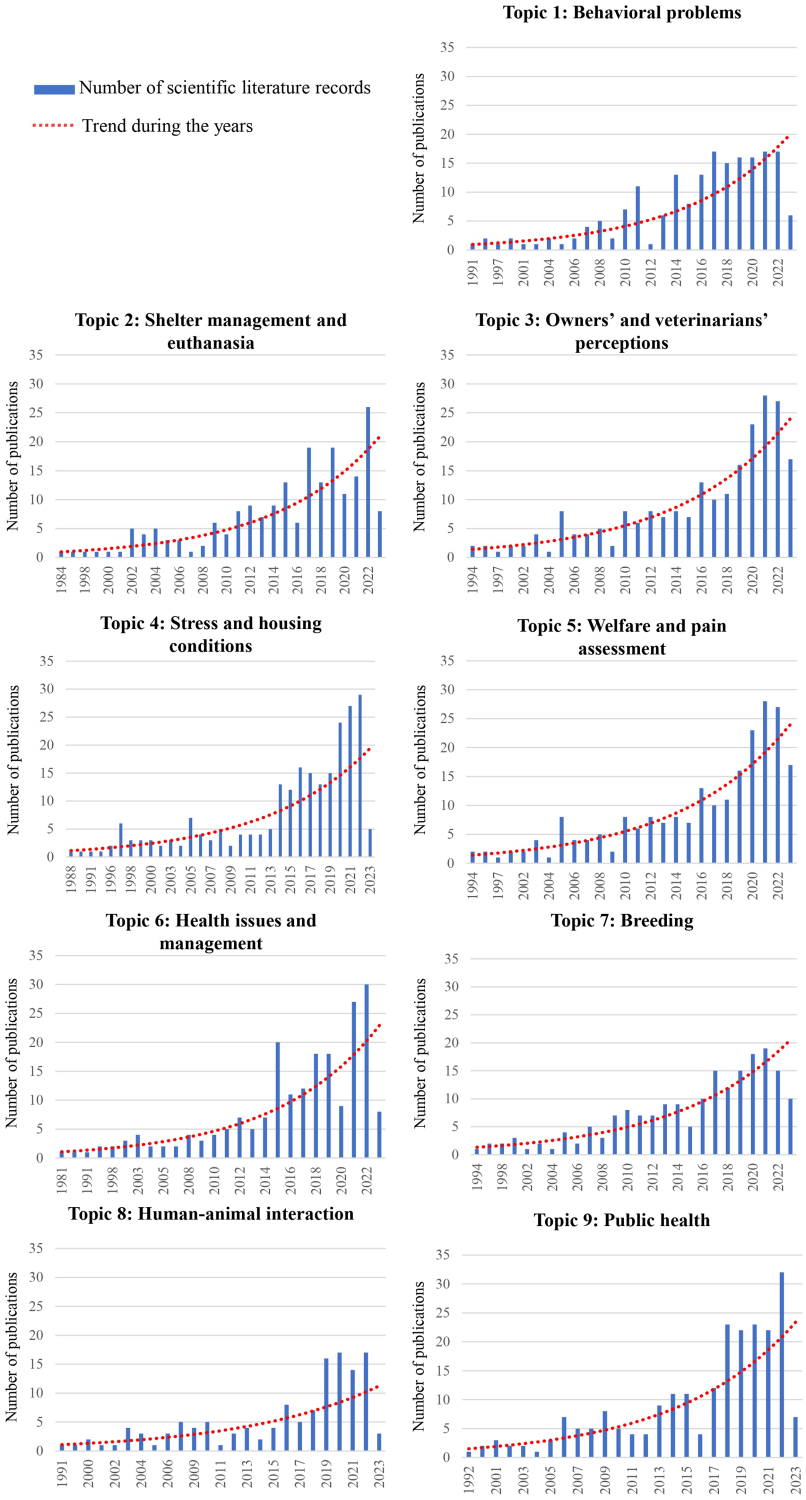
Graphs of the 1,775 records’ distribution over the years 1980–2023 for each topic. The blue lines indicate the number of scientific literature records per year and the dashed lines indicate the trendline for the whole timespan.

Figure 7 shows the 9 topics with the attributed names, and their first 15 words. The most consistent topic was the one named “Stress and housing conditions” (topic 4) followed by topic 9 (“Public health”), and topic 3 (“Owners’ and veterinarians’ perceptions”) with a number of records of 235, 228, and 226, respectively. Following closely behind were Topic 6 (“Health issues and management”) with 208 records and Topic 2 (“Shelter management and euthanasia”) with 201 records. Topic 8 (“Human-animal interaction”) had the lowest number of records published, with only 132 records. The results of the trend analysis for the period 1980 to 2023 were represented in graphs for each topic in Figure 8. A trendline showed that for all the topics there was a significant increase in the number of records, especially after 2010. Most of the records for every topic were published between the years 2019–2022. According to the graphs, topic 6 showed a steady trend in the number of published records from the year 2017–2022.

## 4. Discussions

In this study, we applied text mining analysis and topic modeling to extract valuable insight from a vast body of scientific literature allowing us to examine various topics in the field of companion animal welfare and identify any gaps in the current knowledge. Despite reaching some consensus on an applied definition of welfare, ongoing academic debate continues. Additionally, the political relevance of animal welfare science is strongly based on societal concerns regarding how animals are treated. Animal welfare is seen as a ‘new science’ by many and the development of companion animal welfare science is considered even newer (37). The application of text mining and topic analysis techniques to the cats’ and dogs’ welfare literature has therefore enabled a deep analysis of the research conducted over the past 40 years enhancing our understanding of the subject. The topics that emerged from the topic analysis were expected, particularly those relating to behavioral problems, housing conditions, and health, since those are the three areas that are well-developed in veterinary medicine and husbandry. However, topics such as human-animal interaction and the perception of welfare by owners and veterinarians also surfaced in recent years of research, reflecting the important role of humans and their relationship with animals in the concept of animal welfare. The findings obtained using this machine learning technique confirm also the multidisciplinary of animal welfare topics.

There has been an augmented increase in the number of records in the last 40 years, particularly after the 2000s. The lower number of records found in 2023 is because the search was carried out in May 2023, so the number of records published online was still limited, but an upward trend is expected. This upward trend of publication reflects the development of animal welfare science. The term animal welfare began to be used in 1947 (38), but it was only in the 1990s that it started to be considered a measurable scientific term (39). As it was stated above animal welfare on farm animals has been stricter and more well-regulated than in the case of cats and dogs but despite the delay, it became imperative to ensure the care of dogs and cats in a more secure legal framework (24). Since the early 2000s, countries such as Germany, Austria, and the United Kingdom began to strengthen their state legislation with laws that further shielded the protection of pets (29). In 2004, Italy introduced “Law 189,” which aimed to prevent the abuse of animals and specifically addressed their involvement in underground fights or unapproved contests. Regulation must be based on research, so more funds for animal welfare were available generating more publications. As expected from the descriptive statistics, Europe was the dominant region with the United Kingdom, Italy, Spain, Germany, and Austria as the major geographical areas of origin for records on cats’ and dogs’ welfare. This reflects the region’s pioneering and driving role in the field of animal protection and welfare promotion. North America followed in terms of the number of published records, and it is reasonable considering the large population and research opportunities, especially in the United States where legislation is weak and many pet welfare issues have been highlighted (13).

The most frequent words were “shelter,” “behavior,” “owner,” “adopt” and “stress” (with higher TF-IDF weight). The word “shelter,” as the most frequent word, is not surprising as it may reflect the major problems of relinquishment and overpopulation of shelter animals that compromise the overall welfare of cats and dogs (40–42). It is also not a coincidence that the word “shelter” was associated with euthanasia. In fact, when animals are relinquished, they may be reclaimed, adopted, remain in shelters until they die, or be euthanized (43). Animal welfare groups are striving to reduce euthanasia rates, and many shelters around the world have adopted no-kill policies for adoptable animals as part of their mission. In countries, such as Italy, where a no-kill policy is in place, animals can remain in shelters until they die naturally, and euthanasia can only be used for dogs and cats that are dangerous or have a terminal illness or a health condition that makes life painful (44, 45). A no-kill policy can also have negative aspects. It can be costly and space intensive, leading to chronic overcrowding in shelters and compromising welfare standards. This is why other countries, such as the United States, use euthanasia. The decision to euthanize a shelter animal is influenced by a number of factors, including the animal’s health and any behavioral problems (46). In addition, many shelters face the harsh reality of limited space and funding, which often forces them to make the difficult decision to euthanize animals in order to accommodate new arrivals (47). According to Shelter Animals Count database, a total of 325,301 cats and dogs were euthanized in the US shelters in 2019, with euthanasia being the final outcome for the 11.5% of cats and 6.9% of dogs (20). After 2019, the number of animals relinquished decreased by 16%, although the COVID19 pandemic led to a slight increase in the number of abandoned dogs and cats in 2022 compared to 2020 (48). Interestingly, factors such as age group and coat color have been found to play a role in shelter dog euthanasia decisions (49). The term “behavior” was quite frequent as animal “behavior” has been extensively studied and analyzed in various situations within the field of welfare, especially as a parameter of assessment (50–52). The words “owner” and “adopt” had also a higher probability because they can both be linked with the word shelter. The word “owner” comprises the perspective of the human in relation to the well-being of their companion animal and how they engage and interact with it. Finally, it is not surprising that the word “stress” was often used in the literature, as for a while stress-related responses have been used as indicators of poor quality of welfare.

The LDA analysis has identified nine different topics highlighting the diverse aspects of welfare, ranging from health to behavioral problems, pain, and management. This involves studying and connecting the different biological components, including physical and psychological factors, that together determine the level of welfare. This amplifies that the approach to welfare encompasses multiple disciplines and that the concept of welfare itself is broad and challenging to categorize (53). The topics that emerged align well with the four principles of “Good Feeding,” “Good Housing,” “Good Health,” and “Appropriate Behavior” outlined in the Welfare Quality protocol (54). Topics 2 and 4 can be classified under the principle of “Good Housing” while topics 5, 6, 7, and 9 fall under the category of “Good Health.” Topics 1, 3, and 8 can be associated with the principle of “Appropriate Behavior.” It is worth noting that no specific topic was found to directly address the concept of “Good Feeding.” Clearly, some aspects have been studied more than others and some of them earlier than others, reflecting also whether this argument has been seen as an aspect possibly related to welfare by the authors, which have used in their text the word “welfare.”

As previously mentioned, the topic named “Stress and housing conditions” (topic 4) was that one with more scientific literature records. Stress has indeed gained significant importance as a topic due to its pervasive impact on welfare and this topic comprises the effect of housing conditions under the influence of the “good housing” principle in the stress behavior of cats and dogs. Terms such as “environmental enrichment” and “cortisol levels” are often observed in the research studies which entered this topic. It has been shown that an unenriched environment and inappropriate management by staff can lead to a low quality of life and compromise the welfare of dogs sheltered for long period (50). The second topic for number of records was “Public health” (topic 9). It was noted that this topic encloses subjects such as vaccination and management and represents a very well-researched topic through the years. Indeed, within 20 years, vaccination became an act of veterinary science that should be considered an individualized medicine, adjusted to the needs of each pet. Vaccination has been provided as preventive medicine, being part of an annual health check-up visit (55), and was also as a tool to preserve public health from zoonoses such as leptospirosis and rabies, which are common in stray dogs (56–58). Public health recently also raises concerns about the population of free-roaming animals and the overcrowded spaces in shelters (59, 60). In fact, neglected zoonotic diseases such as rabies and echinococcosis are transmitted at the stray-dog-human interface, particularly in low to middle-income countries (58). Another topic that was found important and is contained in the “Good Housing” principle and also related to public health is the topic named “Shelter management and euthanasia” (topic 2). As mentioned above, the shelter population of cats and dogs is rapidly increasing. This statement can be justified by the words “population,” “number” and “increase” which were found to be associated with the current topic. The studies contained in this topic could also be considered under the umbrella of “one health” and “one welfare” approaches. It is indeed important to rise the welfare of the animals to also enhance human and planet well-being.

As expected, numerous published records, regarding the topic “Owners’ and veterinarians’ perception” (topic 3), have surfaced through the years. Records related to this topic are usually based on questionnaires and surveys that are given to pet owners and veterinarians to express their opinion on different matters of welfare such as during vet visits (61–63) and the management of pet home (64). This topic included many records (*n* = 228) in line with the use of questionnaires in veterinary and animal sciences. In the case of pet welfare, the use of online surveys and telephone and face-to-face interviews has been useful to understand in depth the perception of owners and vets on several welfare-related topics, including cosmetic surgery (65) and pet management, namely training for car drives (66, 67) and veterinary examinations. For instance, Park et al. (68) clarified the relationship between American dog owner characteristics and willingness to seek veterinary care, while in their review La Vallee et al. (69) listed cost, accessibility, veterinarian-client communication barriers, and lack of client education as the most common barriers to veterinary care. Education of owners was also pointed out by Park et al. as a crucial point to improve pet welfare (68).

The research topics named “Behavioral problems” (topic 1) and “Human-animal interaction” (topic 8) had a rise in the number of studies after 2010. This could be because behavioral medicine is a quite novel branch in veterinary medicine, so behavioral problems have started to be diagnosed accurately only in the last decades. A meta-analysis comparing the reasons for dogs being surrendered to shelters revealed that behavioral problems were the most common reason in eight out of nine studies. The reported frequency of relinquishment due to behavioral problems in a retrospective study by Jensen et al. ranged from 11 to 34% (70). The word “training” was associated with this topic and that is because training is a method of managing behavioral problems. A study conducted by Salman et al. (71), showed that owners of dogs facing behavioral problems are more inclined to seek training as a potential solution to address these issues and often have higher expectations for dogs that are perceived as “trained” compared to those considered “untrained.” Pet ownership and the emotional attachment to pets are influential factors that can directly contribute to improved health and emotional well-being of pet owners (72). “Human-animal interaction” is a growing topic that centers around the bond and connection between pet owners and their animals. Studies conducted among American adults (73) and Israeli adults (74) reveal that an increasing number of pet owners consider their dogs or cats as integral members of their family. Words such as “impact” and “social” reflect the influence of pet ownership. Indeed, evidence from epidemiological and psychological studies suggests that pet ownership is associated with several positive health benefits for pet owners (75) and promotes social interaction (76, 77).

“Breeding” (topic 7) and “Welfare and pain assessment” (topic 5) were also studied mostly in the last decade. This could be firstly because selective breeding has become a welfare concern only recently, leading to the ban of the breeding of specific brachycephalic breeds in North European countries. Secondly, because commercial breeding of cats and dogs has been under-regulated worldwide, with a very limited number of studies focusing on the welfare of cats and dogs used for breeding (30). The topic “Welfare and pain assessment” did not contain many records (only 166); however, a higher number of studies was recently published probably due to the fact that the need to objectively measure welfare and pain is quite recent in the literature. A significant amount of research has focused on animal welfare problems, including the development of assessment methods for different environments (53). A scoping review published in 2021 found only a few studies focusing on the welfare and quality of life assessment of shelter dogs and all of them were published not earlier than 2010 (78). The first pain scale based on facial expression in cats was also published only in 2019 (79). The need for more studies aiming at identifying thresholds and aggregation methods to carry out risk analysis in animal welfare was pointed out by the European Food Safety Agency-EFSA (30).

The statistical approach was useful also to highlight other fields not investigated so far in relation to welfare. It is worth noting, indeed, the lack of knowledge and research found to exist about the principle of “Good Feeding.” This could be due to the fact the word “welfare” was not used in the studies focusing on pet nutrition, as nutritionists do not see these two fields of research as interlinked. Another explication could be the fact that pets have usually access to food, so they rarely suffer from prolonged hunger, and consequently, this has not been seen as a welfare issue. On this matter, it is instead important to increase the research on appropriate feeding, which does not only mean offering a diet that covers the energetic requirement but also which meets the behavioral needs of our companion animals, also preventing obesity. Lund et al. (80) found out that 35% of household cats in the United States are obese and according to the Vet Charity for Pets in Need (PDSA) reports, veterinarians in the United Kingdom have witnessed an increase in pet obesity the recent years (81). More research on this topic should be consequently recommended. It should also be noted that there was a lack of research on the welfare of dogs and cats kept as laboratory animals. Dogs and cats have long been utilized in biomedical research due to their anatomical, physiological, and disease-response similarities to humans (82). The welfare considerations for laboratory dogs and cats are fundamentally the same as those for pets, even though the underlying motivations for these decisions may vary. Concerns for animal welfare and advancements in veterinary practices are collectively driving the exploration of alternative approaches to enhance the welfare of animals in laboratory settings among farm and pet animals (83). Furthermore, a less-explored topic was the positive welfare approach. The traditional approach to animal welfare was that negative physical or mental experiences should be minimized, while advances in the understanding of animals with the evolution in societal views have led to the gradual inclusion of positive experiences into definitions of “animal happiness” (84). So, it is increasingly acknowledged that considering only the negative aspects of animal welfare is not enough and by disregarding the positive aspects, there is less recognition of important factors related to animal behavior, physiology, and the considerations that owners naturally consider. These considerations include the animals’ preferences, and their overall quality of life (85).

Our findings need to be interpreted with caution as several limitations should be considered as typical of the statistical method applied. Firstly, the search is strictly related to the keywords, so, although the search strings for entry into the Scopus® search were discussed in detail within the research team, some synonyms (e.g., “feline,” “canine,” and “well-being”) have not been included and consequently our results may be underestimating the relative literature. Similarly, the search was limited to a single database, namely Scopus®, and thus some records published in journals not included in it may have been missed. Moreover, certain predetermined parameters were set before starting the research, including the restriction to English-only language records. Additionally, the adopted screening criteria may have resulted in a partial reduction in the number of records that were thoroughly analyzed. It is important to note that in this method of analysis, the 1,775 records were not read in their entirety but only the titles and abstracts were taken into consideration. Nevertheless, it is important to emphasize that the technique used might not have revealed other topics that could be more recent or of lesser scientific interest. Finally, only text mining and topic analysis were performed, but other statistical analysis, such as text mining on multi-word phrases and cluster analysis, have not been performed.

Notwithstanding those limitations, this review extensively examined the literature concerning the welfare of cats and dogs and it successfully identified the research areas that have been extensively studied as well as the subjects that require further scientific evidence. Consequently, this review contributes as a valuable resource for future researchers, providing a foundation for further research in less-explored areas.

## 5. Conclusion

This review analyzed the literature related to the welfare of dogs and cats using machine learning methods. It found that dog and cat welfare is a growing field and that at least 9 different topics related to pet welfare could be identified as areas of research that have been studied to a greater or lesser extent over the past 40 years. There is a lack of research in areas such as optimal feeding practices, positive welfare, and the welfare of cats and dogs used as laboratory animals. Given that future legislation to protect the welfare of cats and dogs will need to be based on research, further studies are recommended to enhance our understanding of the welfare needs of companion animals and how to ensure positive welfare for them. More studies and reviews addressing companion animal welfare topics are therefore recommended.

## Author contributions

CA: Data curation, Formal analysis, Investigation, Methodology, Writing – original draft. BB: Conceptualization, Data curation, Formal analysis, Investigation, Methodology, Resources, Writing – original draft. MZ: Conceptualization, Funding acquisition, Investigation, Methodology, Resources, Writing – review & editing. MF: Conceptualization, Funding acquisition, Methodology, Resources, Writing – review & editing. NM: Investigation, Writing – review & editing. AlP: Investigation, Writing – original draft. AmP: Writing – review & editing. BP: Conceptualization, Funding acquisition, Investigation, Methodology, Project administration, Supervision, Writing – original draft, Writing – review & editing.

## Footnotes

^1^ www.wordclouds.com
